# Paraquat Induces Epithelial-Mesenchymal Transition-Like Cellular Response Resulting in Fibrogenesis and the Prevention of Apoptosis in Human Pulmonary Epithelial Cells

**DOI:** 10.1371/journal.pone.0120192

**Published:** 2015-03-23

**Authors:** Atsushi Yamada, Toshihiko Aki, Kana Unuma, Takeshi Funakoshi, Koichi Uemura

**Affiliations:** Department of Forensic Medicine, Graduate School of Medical and Dental Sciences, Tokyo Medical and Dental University, Tokyo Japan; Northwestern University, UNITED STATES

## Abstract

The aim of this study is to investigate the molecular mechanisms underlying delayed progressive pulmonary fibrosis, a characteristic of subacute paraquat (PQ) poisoning. Epithelial-mesenchymal transition (EMT) has been proposed as a cause of organ fibrosis, and transforming growth factor-β (TGF-β) is suggested to be a powerful mediator of EMT. We thus examined the possibility that EMT is involved in pulmonary fibrosis during PQ poisoning using A549 human alveolar epithelial cells *in vitro*. The cells were treated with various concentrations of PQ (0–500 μM) for 2–12 days. Short-term (2 days) high-dose (>100 μM) treatments with PQ induced cell death accompanied by the activation of caspase9 as well as a decrease in E-cadherin (an epithelial cell marker), suggesting apoptotic cell death with the features of anoikis (cell death due to the loss of cell-cell adhesion). In contrast, long-term (6–12 days) low-dose (30 μM) treatments with PQ resulted in a transformation into spindle-shaped mesenchymal-like cells with a decrease of E-cadherin as well as an increase of α-smooth muscle actin (α-SMA). The mesenchymal-like cells also secreted the extracellular matrix (ECM) protein fibronectin into the culture medium. The administration of a TGF-β1 receptor antagonist, SB431542, almost completely attenuated the mesenchymal transformation as well as fibronectin secretion, suggesting a crucial role of TGF-β1 in EMT-like cellular response and subsequent fibrogenesis. It is noteworthy that despite the suppression of EMT-fibrogenesis, apoptotic death was observed in cells treated with PQ+SB431542. EMT-like cellular response and subsequent fibrogenesis were also observed in normal human bronchial epithelial (NHBE) cells exposed to PQ in a TGF-β1-dependent manner. Taken together, our experimental model reflects well the etiology of PQ poisoning in human and shows the involvement of EMT-like cellular response in both fibrogenesis and resistance to cell death during subacute PQ poisoning of pulmonary epithelial cells.

## Introduction

Although paraquat (PQ, *1*,*1'*-dimethyl-*4*,*4'*-bipyridinium) is a highly toxic compound, and its use is rigorously restricted in many industrialized countries including the United States and the members of European Union, it is still widely used as a herbicide in many developing countries around the world. Accidental as well as intentional ingestion of PQ causes fatal poisoning in human beings through the generation of reactive oxygen species (ROS) that cause cellular damage such as lipid peroxidation, mitochondrial damage and apoptosis [1]. Although PQ distributes in multiple tissues, it accumulates mainly in the lung and kidney. In the lung, PQ accumulates at particularly high levels in Clara cells, as well as in alveolar type I and II epithelial cells [1, 2]. The lung injury caused by PQ is characterized by epithelial cell destruction, followed by secondary alveolitis that is defined by pulmonary edema and inflammation. Delayed progressive pulmonary fibrosis is the most characteristic feature of subacute PQ poisoning, which occurs over a period from days to weeks after PQ ingestion [1]. Although PQ-induced pulmonary fibrosis is associated with high mortality, the molecular mechanism of its toxicity and effective antidotes are not established to date.

Pulmonary fibrosis has been recognized to result from the activation (*e*.*g*. proliferation) of fibroblasts and the subsequent accumulation of extracellular matrix (ECM) proteins. Indeed, the maturation of profibroblasts into fibroblasts in the lung is observed in the days to weeks following the administration of PQ to rats [3]. However, recent research has revealed other mechanisms involved in pulmonary fibrosis: 1) bone marrow cells can be a source of fibroblasts, and the recruitment of bone marrow-derived fibroblasts into fibroblastic lesions is observed in several cases of drug intoxication in the lung [4, 5]; and 2) lung epithelial cells can also be a source of fibroblasts after the acquisition of mesenchymal phenotypes through epithelial-mesenchymal transition (EMT). EMT is a process during which epithelial cells are converted to mesenchymal cells such as myofibroblasts [6]. EMT is accompanied by a decrease in epithelial cell markers such as E-cadherin as well as an increase in mesenchymal cell makers such as α-smooth muscle actin (α-SMA). Consequently these cells attain the capacity to secrete ECM proteins. Recent studies suggest the potential contributions of EMT in lung fibrosis [7, 8].

EMT is proposed to play an important role in maintaining cellular homeostasis in injured organs and tumor microenvironment. Inflammation, oxidative stress and hypoxia are the potential triggers of EMT by which multiple signaling molecules such as transforming growth factor-β1 (TGF-β1) and ROS are generated, after which the activation of hypoxia-inducible factor-1 (HIF-1), Snail, Twist, and ZEB1 transcription factors takes place [9]. The pathophysiological role of EMT involves the initiation of tissue repair via the production of ECM proteins, although excessive EMT-fibrogenesis may lead to organ failure due to excessive fibrosis. EMT is also implicated in tumor progression by promoting tumor metastasis. Loss of appropriate cell-cell adhesion of the epithelium generally induces a specific type of apoptotic cell death known as anoikis [10, 11]. Epithelium-derived tumors escape anoikis by acquiring a mesenchymal-phenotype through EMT and move toward new metastatic lesions [12].

Recent reports have suggested that several substances such as bleomycin [13], hydrogen peroxide [14], and multi-walled carbon nanotubes [15] could be inducers of EMT in lung injury. A role for TGF-β1 and HIF-1 in animal models of lung injury caused by PQ poisoning has also been indicated [16, 17]. We hypothesized that EMT has also important roles in both cell death and fibrogenesis in PQ-induced lung injury, and, therefore, we explored the relationships between PQ poisoning, EMT and cell death using A549 human alveolar epithelial carcinoma cells and NHBE normal human bronchial epithelial cells *in vitro*.

## Materials and Methods

### Materials

Paraquat dichloride was purchased from SIGMA-ALDRICH (St. Louis, MO, USA). The TGF-β1 receptor antagonist SB431542 was also purchased from SIGMA-ALDRICH. Antibodies used for Western blot analysis and fluorescence immunostaining were anti-E-cadherin (610181, BD Biosciences, San Jose, CA, USA), anti-α-SMA (A2547, SIGMA-ALDRICH), vimentin (V6630, SIGMA-ALDRICH), myosin11 (ab53219, abcam, Cambridge, UK), anti-caspase-9 (#9502, Cell Signaling Technology, MA, USA), anti-fibronectin (610077, BD Biosciences), anti-TGF-β1 (sc-146, Santa Cruz Biotechnology, Santa Cruz, CA, USA), anti-glyceraldehyde-3-phosphate dehydrogenase (GAPDH) (#MAB374, Millipore, CA, USA), anti-ZEB1 (#3396, Cell Signaling Technology), anti-Twist (sc-15393, Santa Cruz Biotechnology), and anti-Snail (#3879, Cell Signaling Technology).

### Cell culture

A549 human lung adenocarcinoma epithelial cells (RCB0098, provided by the RIKEN BRC through the National Bio-Resource Project of the MEXT, Japan) were cultured in DMEM medium supplemented with 10% fetal bovine serum, 100 U/mL penicillin, and 100 U/mL streptomycin at 37°C in a humidified 5% CO_2_ atmosphere. NHBE normal human bronchial epithelial cells (CC-2540, Lonza, Basel, Switzerland) were cultured in bronchial epithelial cell growth medium (BEGM, CC-3170, Lonza) at 37°C in a humidified 5% CO_2_ atmosphere.

### Measurement of cell viability

Cell viability was assayed by quantifying plasma membrane damage or rupture. Relative activities of lactate dehydrogenase (LDH) liberated from cells due to cell membrane injury were assessed. Following exposure to PQ, LDH activities were determined in the culture medium as well as in cell lysates using an LDH-Cytotoxic test kit (Wako Pure Chemicals, Osaka, Japan). In brief, adherent cells as well as culture supernatants were lysed with 0.2% Tween 20 and examined for their LDH activities. LDH leakage (%) was calculated by dividing LDH activity in the culture supernatants by that in the cell lysates plus in the culture supernatants; sum of the LDH activity in culture supernatant and cell lysate was set as 100% in each assay.

### Western blot analysis

Following exposure to PQ, the cells were detached from the culture dishes with a cell scraper and collected together with floating cells by centrifugation. The collected cells were disrupted using a sonication device (10 s, 3 times; Sonifer 150, Branson, Danbury, CT, USA) in STE buffer [0.32 M sucrose, 10 mM Tris–HCl (pH 7.4), 5 mM EDTA, 50 mM NaF, 2 mM Na_3_VO_4_] containing protease inhibitor cocktail (Complete, Roche, Manheim) on ice. Protein concentrations were determined by the Coomassie Brilliant Blue staining method [18]. Equal amounts of proteins were separated by SDS-PAGE according to the method of Laemmli [19] and transferred to a PVDF membrane. For the analysis of conditioned medium, equal volumes of medium were loaded and separated by SDS-PAGE. After blocking, the membrane was incubated overnight at 4°C with appropriate primary antibodies, washed with TBS-Tween and incubated for 45 min with peroxidase-conjugated anti-rabbit or anti-mouse antibodies (Promega, Madison, WI, USA). Bands were detected using a Western Lightning Chemiluminescence Reagent Plus Kit (PerkinElmer Life Science, Boston, MA, USA), and the intensities of the bands were quantified using an image analyzer (CS analyzer; Atto, Tokyo, Japan).

### Annexin V-FITC/PI staining

To examine whether cell death by PQ was apoptosis or not, annexin V-FITC/propidium iodide (PI) double-staining assay was done according to the manufacturer's protocol (MEBCYTO Apoptosis Kit Annexin V-FITC Kit; MBL, Nagoya, Japan). In brief, cells were detached from dishes by trypsin, incubated with a solution containing annexin V-FITC/PI, mounted on a glass slide, and observed under fluorescence microscopy. To discriminate apoptotic and necrotic cell death, cells incubated with 0.5 M hydrochloric acid (HCl) for 5 minutes at room temperature were used as necrotic cells.

### Quantitative real-time PCR

The expressions of the mRNAs for E-cadherin and α-SMA were analyzed by quantitative real-time PCR (qPCR) using GAPDH as an internal control. In brief, total RNA was prepared from cells using the TRIzol reagent (Invitrogen, Carlsbad, CA, USA), and reverse transcription was performed using SuperScript II reverse transcriptase (Invitrogen). qPCR was performed with the StepOnePlus qPCR System (Applied Biosystems, Foster City, CA, USA) using SYBR green as a fluorescent dye. The PCR conditions were as follows: 95°C for 20 s followed by 40 cycles of 95°C for 3 s and 60°C for 30 s. The primers used were: 5'-ATGCTGATGCCCCCAATACC-3' and 5'-GCTGCTTGGCCTCAAAATCC-3' for E-cadherin; 5'-GACGCCTGCTCCAAGAATTG-3' and 5'-GTCAAGCCGGACACAACGAT-3' for α-SMA; 5'-GGTCGGAGTCAACGGATTTGGTCG-3' and 5'-CCTCCGACGCCTGCTTCACCAC-3' for GAPDH.

### ELISA for TGF-β1

Extracellular levels of TGF-β1 were assessed by enzyme-linked immunosorbent assay (ELISA) according to the manufacturer's protocol (Quantikine ELISA Human TGF-β1 Immunoassay, R&D Systems, Minneapolis, MN, USA). During exposure to 30 μM PQ for 12 days, culture medium was exchanged for new medium every three days, and TGF-β1 levels in conditioned medium (10–12 days with or without PQ exposure) were determined after the removal of cell debris by centrifugation.

### Fluorescence immunostaining

A549 cells grown on cover slips were washed three times with PBS and fixed with 4% paraformaldehyde for 5 min. After treatment with 0.5% Triton X-100 for 5 min, the cells were incubated with appropriate primary antibodies overnight at 4°C. After washing with PBS, secondary antibodies conjugated with a fluorescent dye (Alexa488 for green or Alexa549 for red fluorescence) were added, and the samples were further incubated for 30 min at room temperature. Next, the cells were sealed with Vectashield mounting medium (Vector Laboratories, Burlingame, CA, USA) and observed under a fluorescence microscope.

### Measurement of soluble collagen in culture medium

To detect the secretion of collagen from the cells into the medium, extracellular levels of collagen were assessed by a method based on Sircol dye-binding to collagen using a kit (Sircol Collagen Assay Kit, Biocolor, Belfast, Northern Ireland). During exposure to 30 μM PQ for 12 days, soluble collagen levels in the conditioned medium (10–12 days with or without PQ exposure) were determined.

### Statistical analysis

Pairwise comparisons between means of two groups were performed using a Student t-test. Multiple comparisons were performed using ANOVA. More than two data were subjected to Dunnett’s statistical analysis. The data are expressed as mean ± SD (standard deviation) of at least 3 independent samples. *p* values less than 0.05 were considered to be statistically significant.

## Results

### Apoptotic death of A549 cells after high-dose short-term exposure to PQ

**Fig 1:**
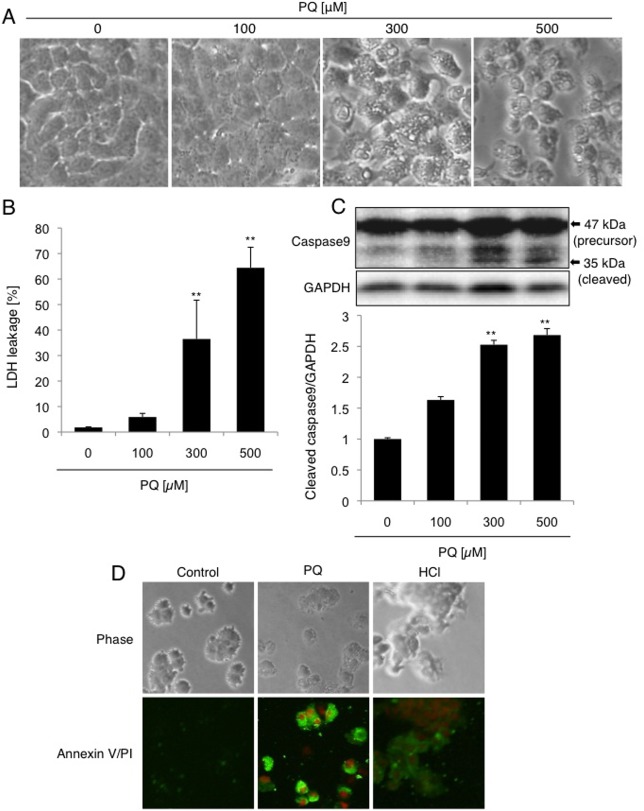
High-dose short-term exposure to PQ induces caspase9 activation and subsequent A549 cell death. (A) Cytomorphology of A549 cells exposed to PQ. Cells were treated with 0, 100, 300, or 500 μM PQ for 2 days and observed under light microscopy. (B) LDH leakage into the medium in PQ-treated cells. The percentages of LDH in the medium were examined after exposure to the indicated concentrations of PQ for 2 days. (C) Activation of caspase9 in PQ-treated (2 days) cells. The cleaved form (35 kDa, indicated by the arrow) of caspase9 was detected by western blot analysis (upper panel). GAPDH served as a loading control (middle panel). Densitometric analysis of band intensities. Levels of cleaved caspase9 relative to GAPDH are shown (mean and SD, n = 4). The value of the control was set to 1. **p < 0.01 versus zero. (D) Phosphatidylserine (PS) exposure in PQ-treated cells. Cells were treated with 500 μM PQ for 2 days and the PS exposure as well as loss of plasma membrane integrity was assessed by staining cells with Annexin V-FITC/PI. Merged images of green (Annexin V-FITC) and red (PI) fluorescences were shown. Cells treated with HCl (0.5 M, 5 minutes) were used as necrotic cells.

We first examined the effects of high-dose (100–500 μM) and short-term (2 days) exposure to PQ on the cytomorphology of A549 cells. The cells were exposed to 0, 100, 300, or 500 μM PQ for 2 days. Cytomorphology was observed under light microscopy: cells showing rounded morphology, aggregation, and flotation in the medium were observed after exposure to 300 or 500 μM PQ, suggesting the induction of cell death by high-dose and short-term exposure to PQ (Fig. 1A). Significant cell death after exposure to 300 and 500 μM PQ was proved by measuring the lactate dehydrogenase (LDH) liberated from the cells due to membrane injury (Fig. 1B). To evaluate whether cell death by PQ was apoptosis or not, caspase9 activation and phosphatidylserine (PS) exposure were examined. After high-dose (300 and 500 μM) exposure to PQ, the cleaved (activated) form of caspase9 and the externalization of PS on cell surface was detected by Western blot analysis and annexin V staining, respectively (Fig. 1C and 1D). Therefore, high-dose exposure to PQ induces apoptotic cell death in A549 cells, as reported previously [20, 21].

### Loss of E-cadherin during A549 cell death by high-dose PQ exposure

**Fig 2:**
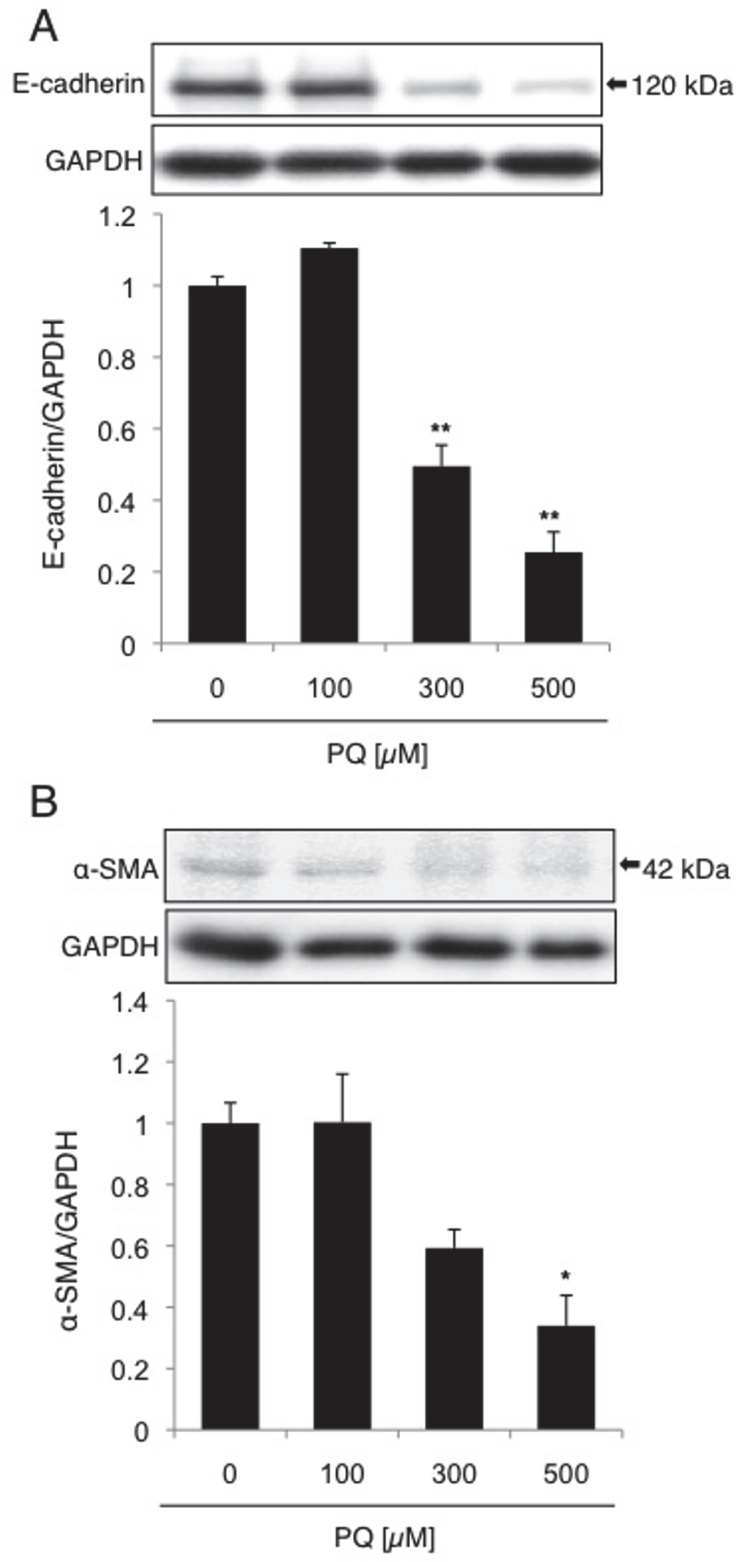
A549 cell death by high-dose short-term PQ treatment is accompanied by a decrease in the epithelial cell marker E-cadherin, but not by an increase in the mesenchymal cell marker α-SMA. A549 cells were treated with 0, 100, 300, or 500 μM PQ for 2 days, and examined for the levels of E-cadherin (A) and α-SMA (B) by Western blot analysis. The levels of E-cadherin and α-SMA were measured by densitometric analysis, and the expression levels of E-cadherin and α-SMA relative to GAPDH are shown (mean and SD, n = 4). The value of the control was set to 1. *p < 0.05, **p < 0.01 versus zero.

We next evaluated whether PQ induces EMT in A549 cells. The cells were exposed to 0, 100, 300, or 500 μM PQ for 2 days, and the expression levels of E-cadherin as well as α-SMA were examined. After high-dose (300 μM PQ as the lowest effective dose) exposure to PQ, a decrease in E-cadherin was observed (Fig. 2A) while a decrease in α-SMA was also detected (Fig. 2B). Loss of E-cadherin is one of the features of anoikis-like apoptotic cell death [22], and decrease of α-SMA during myofibroblast apoptosis have also been reported [23, 24], for example, due to caspase3-mediated proteolysis [23]. Thus, high-dose exposure to PQ induces apoptotic cell death that is accompanied by a decrease in E-cadherin as well as α-SMA, implying that PQ-induced cell death is not associated with EMT-like response, and, therefore, might be anoikis.

#### Low-dose long-term PQ exposure induces EMT-like response in A549 cells

**Fig 3:**
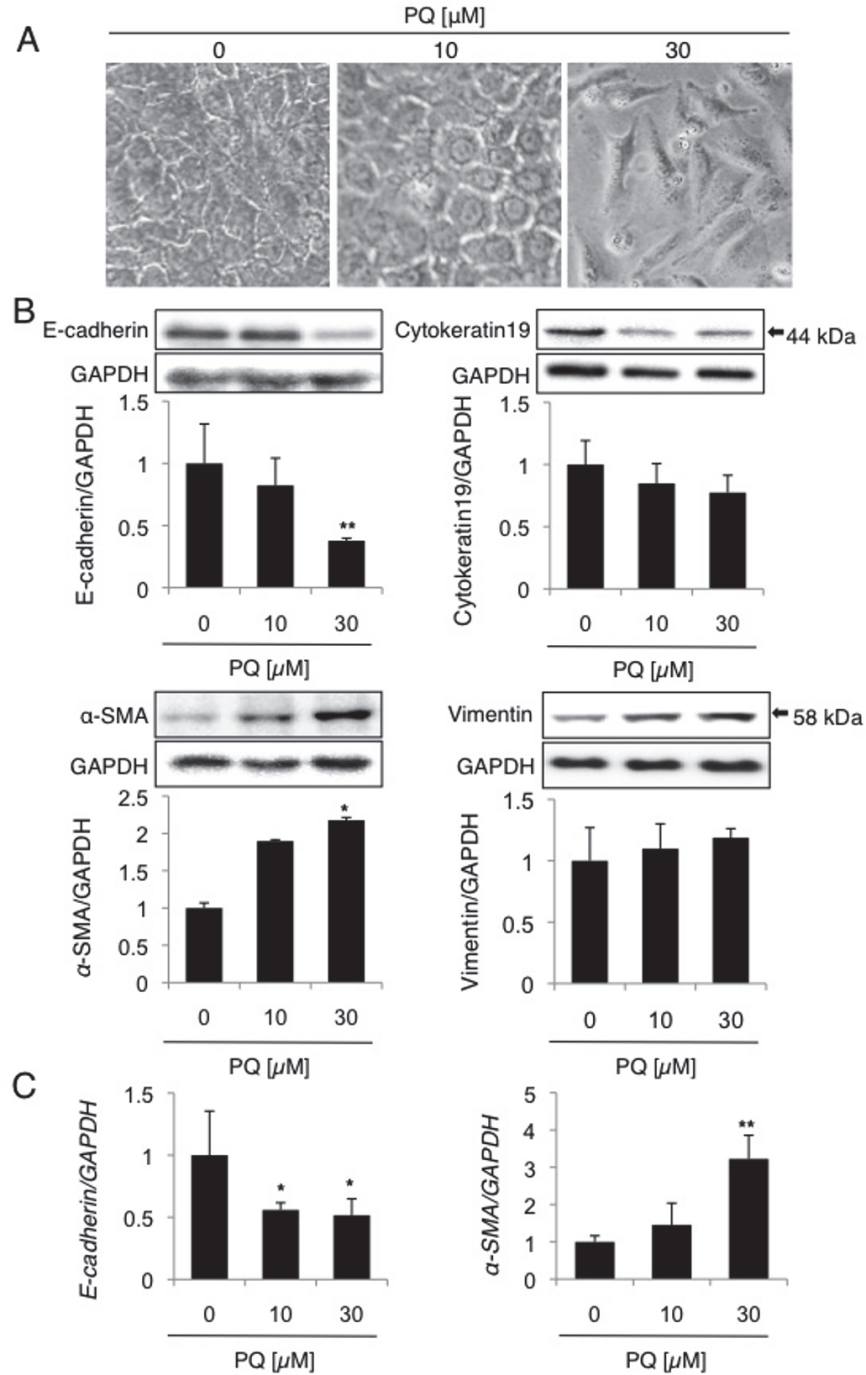
Low-dose long-term exposure to PQ induces both a decrease in E-cadherin and an increase in α-SMA. A549 cells were treated with 0, 10, or 30 μM PQ for 6 days and examined for cell morphology as well as the levels of E-cadherin and α-SMA. (A) Cytomorphology of A549 cells exposed to PQ was observed under light microscopy. (B) The decrease in E-cadherin and the increase in α-SMA proteins in PQ-treated cells. The levels of the E-cadherin and α-SMA proteins were determined by Western blot analysis, and levels of these proteins relative to GAPDH were measured by densitometric analysis (mean and SD, n = 4). (C) The decrease in E-cadherin and the increase in α-SMA mRNAs in PQ-treated cells. The levels of E-cadherin and α-SMA mRNAs were determined by qPCR analysis. The levels of the mRNAs relative to GAPDH are shown (mean and SD, n = 3–4). The value of the control was set to 1. *p < 0.05, **p < 0.01 versus zero.

To investigate further whether PQ induces EMT-like response in A549 cells, cells were exposed to low doses (0, 10, or 30 μM) of PQ for 6 days. Cells not exposed to PQ showed the cobblestone-like appearance characteristic of epithelial cells (Fig. 3A). In contrast, cells exposed to 30 μM PQ showed a morphological transformation into spindle-shaped mesenchymal-like cells (Fig. 3A). It seems that the cell number is decreased during PQ exposure (Fig. 3A), probably due to the transient attenuation of cell cycle progression during EMT [25, 26]. Western blot analysis demonstrated that the expressions of E-cadherin and α-SMA are significantly decreased and increased, respectively, after exposure to 30 μM PQ (Fig. 3B). Another EMT markers, cytokeratin19 (an epithelial marker) and vimentin (a mesenchymal marker) also showed tendencies to decrease and increase, respectively, after exposure to 30 μM PQ (Fig. 3B). RT-PCR analysis also demonstrated that the levels of E-cadherin and α-SMA mRNAs were significantly decreased and increased, respectively, after exposure to 30 μM PQ (Fig. 3C). Collectively, we conclude that low-dose (30 μM) long-term (6 days) PQ exposure induces EMT-like cellular response in A549 cells.

#### Low-dose long-term PQ exposure induces nuclear translocation of EMT-inducing transcription factors in A549 cells

**Fig 4:**
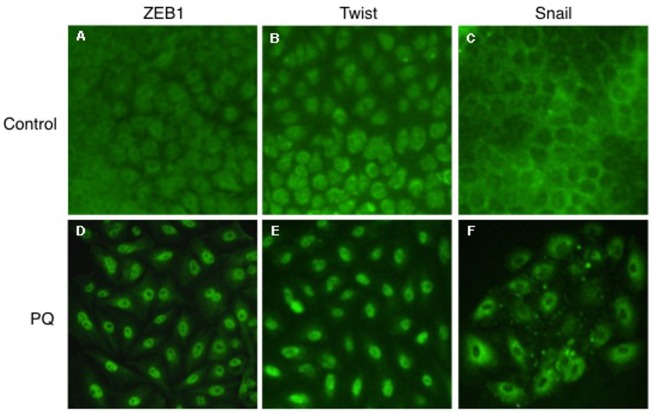
Low-dose long-term exposure to PQ causes nuclear translocation of EMT-inducing transcription factors. Following 6 days of exposure to 30 μM PQ, A549 cells were treated with (D-F) or without (A-C) 30 μM PQ for 6 days. Immunofluorescence analysis was performed using anti-ZEB1, anti-Twist, and anti-Snail antibodies.

Given the evidences of EMT-like cellular response (Fig. 3), we examined whether EMT-inducing transcription factors, ZEB1, Twist, and Snail, were activated during low-dose long-term PQ exposure in A549 cells. Immunofluorescence analysis showed that ZEB1 and Twist were localized to nucleus after exposure to 30 μM PQ for 6 days (Fig. 4). Although altered subcellular localization of Snail was also observed in the cells during PQ exposure, it was localized in the perinuclear region even after PQ exposure (Fig. 4). These results suggest that at least two EMT-inducing transcription factors, ZEB1 and Twist, are activated after exposure to 30 μM PQ for 6 days.

#### Low-dose long-term PQ exposure induces the secretion of fibronectin via TGF-β1 signaling in A549 cells

**Fig 5:**
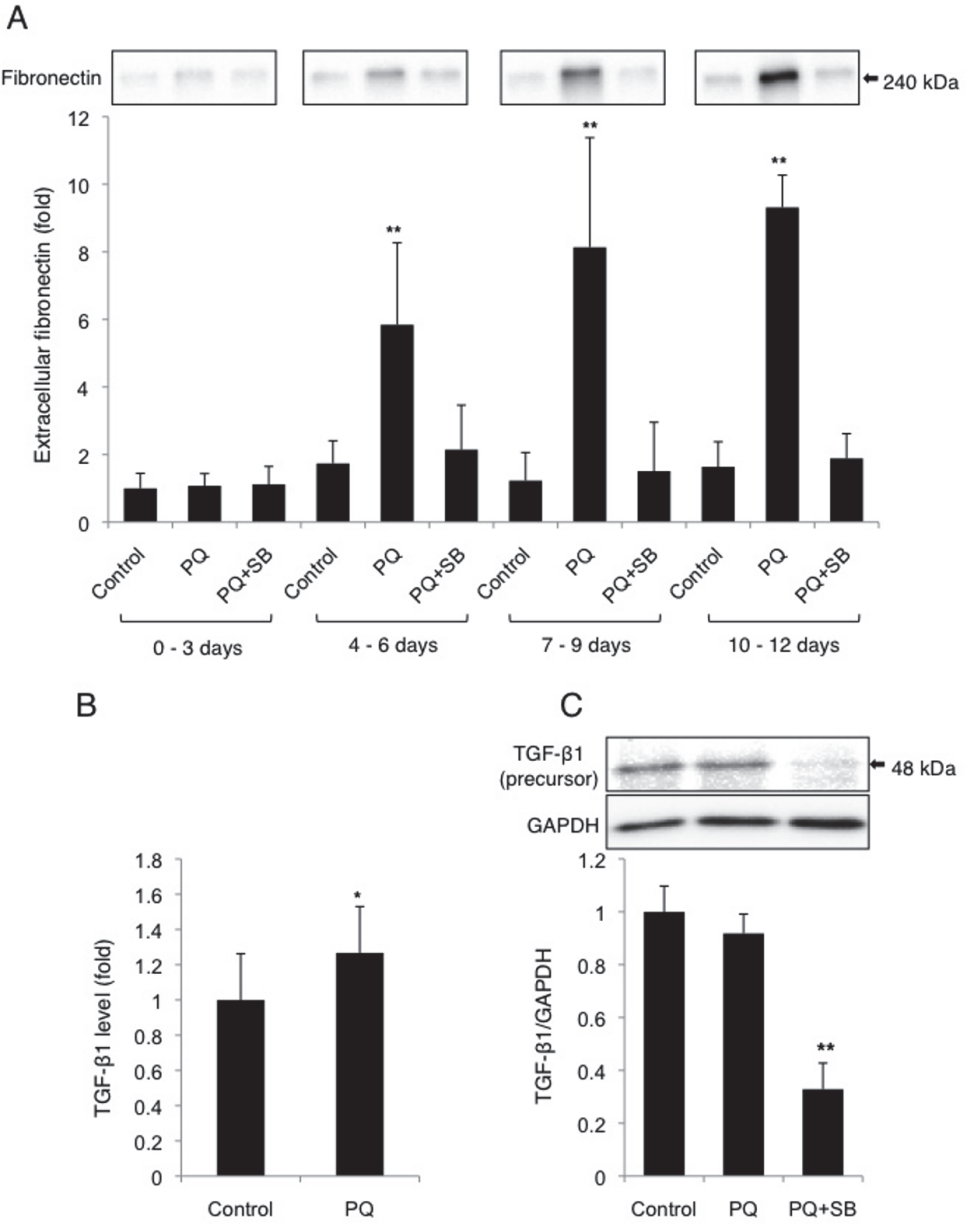
Secretion of fibronectin and its inhibition by a TGF-β1 receptor antagonist from A549 cells treated with low-dose PQ. (A) Low-dose PQ exposure induces the secretion of fibronectin from A549 cells in a TGF-β1 receptor-dependent manner. Cells were exposed to 30 μM PQ with or without 10 μM SB431542, a TGF-β1 receptor antagonist, for 12 days, and the conditioned culture medium was analyzed for its fibronectin level by Western blot analysis. The same volumes of medium were applied to each lane. Fibronectin levels as determined by densitometric analysis are shown (mean and SD, n = 4). The mean fibronectin level in 0–3 days samples was set to 1. (B) Low-dose PQ exposure increases the levels of extracellular TGF-β1. TGF-β1 levels in the conditioned culture medium (10–12 days with or without 30 μM PQ) were examined by ELISA (mean and SD, n = 4). (C) SB431542 down-regulates the expression of the TGF-β1 precursor. No increase in TGF-β1 expression was detected following PQ exposure. Cells were exposed to 30 μM PQ with or without 10 μM SB431542 for 12 days, and examined for the level of the TGF-β1 precursor protein by Western blot analysis. The level of the protein relative to GAPDH is shown (mean and SD, n = 3). The value of the control was set to 1. *p < 0.05, **p < 0.01 versus zero.

It has been suggested that EMT plays important roles in tissue fibrogenesis, and we have shown that low-dose PQ induces EMT-like response in A549 cells (Figs. 3 and 4). We, therefore, evaluated whether A549 cells attain the capacity to secrete ECM proteins after EMT-like response by low-dose PQ exposure. We first examined the extracellular levels of fibronectin, an ECM protein. Cells were exposed to 30 μM PQ for 12 days with or without SB431542, a TGF-β1 receptor antagonist. SB431542 was included in the medium at a final concentration of 10 μM, since it has been shown that 10 μM SB431542 effectively inhibits TGF-β1 receptor signaling without affecting other signaling pathways [27, 28]. The culture medium was exchanged for new medium every three days, and the fibronectin levels in the conditioned medium were examined following the removal of cell debris by centrifugation. During exposure to 30 μM PQ for 12 days, extracellular fibronectin levels began to increase after 4–6 days of PQ exposure (Fig. 5A). Extracellular fibronectin levels were increased in a time-dependent manner during PQ exposure, and reached a level about 10-fold higher than that of the control group after 10–12 days of exposure (Fig. 5A). SB431542 almost completely attenuated the secretion of fibronectin, suggesting the involvement of TGF-β1 signaling in PQ-induced fibrogenesis in A549 cells (Fig. 5A). Concordant with the observation of TGF-β1-dependent fibronectin secretion in PQ-exposed cells, increased levels of extracellular TGF-β1 were observed after 10–12 days of PQ exposure, indicating increased secretion of TGF-β1 from PQ-treated cells (Fig. 5B). The levels of the intracellular precursor form of TGF-β1 did not increase even after 6 days of exposure to 30 μM PQ (Fig. 5C). However, SB431542 severely attenuated the expression of the TGF-β1 precursor (Fig. 5C). Thus, SB431542 indeed attenuates TGF-β1 signaling in PQ-exposed cells, which results in the suppression of fibronectin secretion from the cells.

#### EMT-like cellular response is involved in the deposition of fibronectin on PQ-treated A549 cells

**Fig 6:**
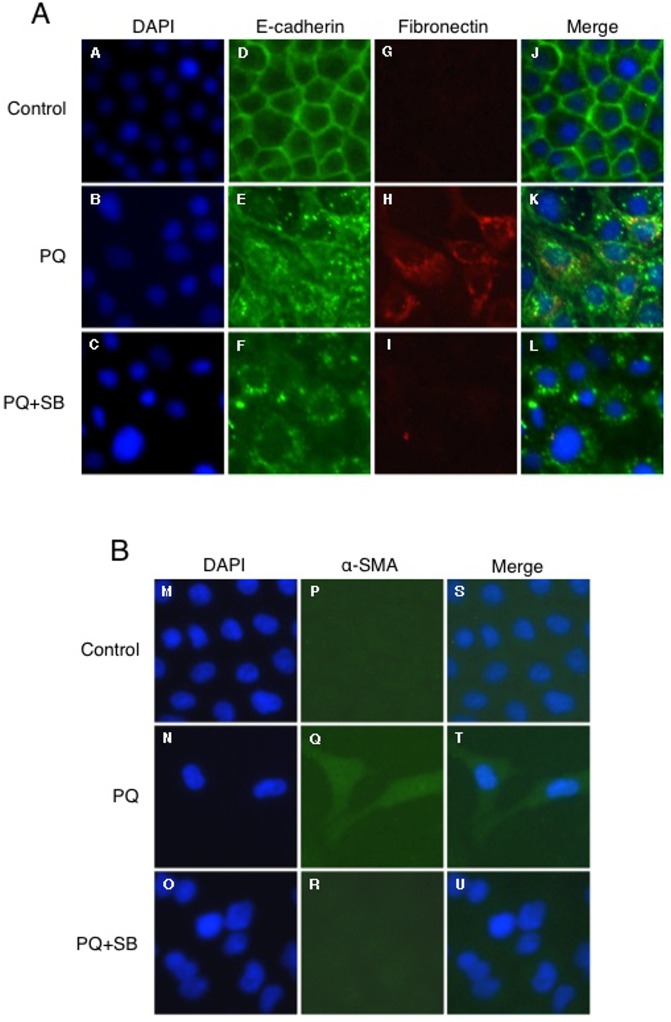
Immunofluorescence analysis of E-cadherin, fibronectin, and α-SMA in A549 cells treated with low-dose PQ for 6 days. Following 6 days of exposure to 30 μM PQ with or without SB431542, the cells were double-stained with anti-E-cadherin (D-F; green) and anti-fibronectin (G-I; red), or stained with anti-α-SMA (P-R; green), and observed under fluorescence microscopy. Nuclei were counterstained with DAPI (A-C and M-O; blue). Merged images are also shown (J-L and S-U).

**Fig 7:**
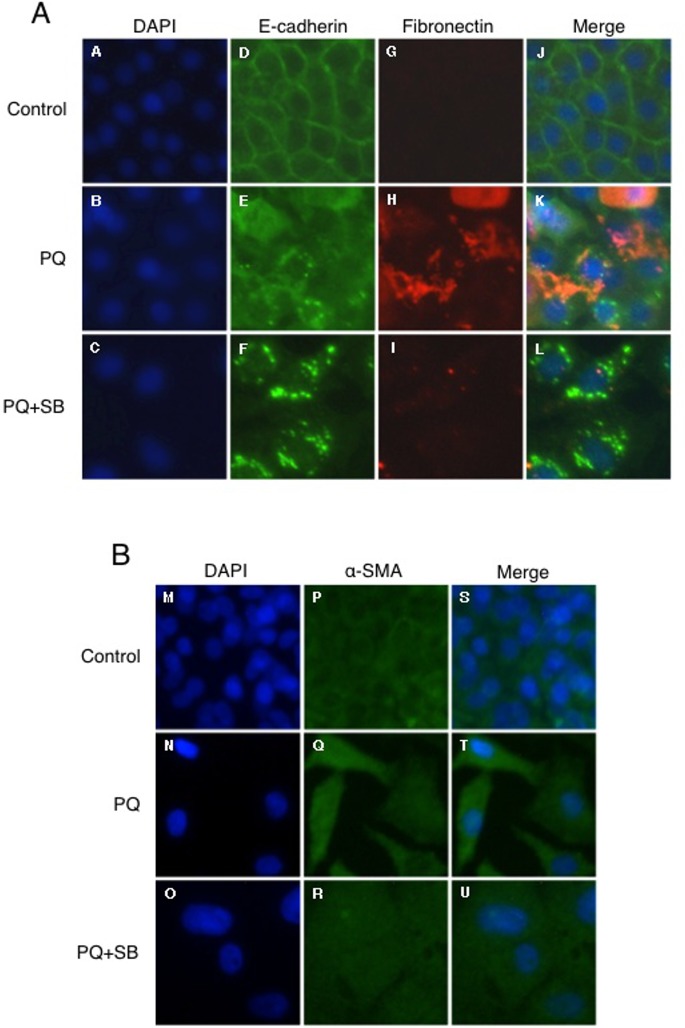
Immunofluorescence analysis of E-cadherin, fibronectin, and α-SMA in A549 cells treated with low-dose PQ for 12 days. Following 12 days of exposure to 30 μM PQ with or without SB431542, the cells were double-stained with anti-E-cadherin (D-F; green) and anti-fibronectin (G-I; red), or stained with anti-α-SMA (P-R; green), and observed under fluorescence microscopy. Nuclei were counterstained with DAPI (A-C and M-O; blue). Merged images are also shown (J-L and S-U).

We next evaluated the relationship between EMT-like response and fibrogenesis in PQ-treated A549 cells. Cells were exposed to 30 μM PQ for 6 days with or without 10 μM SB431542, and subjected to immunofluorescence analysis. The cells were stained with antibodies against E-cadherin and α-SMA to discriminate between epithelial-like and mesenchymal-like cells. E-cadherin localized to the plasma membrane in non-treated cells (control), corresponding to its localization in the adherent junction of epithelial cells (Fig. 6A). In contrast, E-cadherin appeared as cytoplasmic granules in PQ-treated cells (Fig. 6A), suggesting internalization into the cytoplasm for degradation. Strong fibronectin staining was also detected in some PQ-treated cells in which E-cadherin was localized in cytoplasmic granules (Fig. 6A). Greater α-SMA expression was also observed in PQ-treated cells, especially in spindle-shaped cells (Fig. 6B). Interestingly, the spindle-shaped mesenchymal-like morphology, increased α-SMA expression, and increased fibronectin production was not observed in PQ+SB431542-treated cells although the loss of cell surface E-cadherin was observed in these cells as well (Fig. 6A and 6B). Hence, the loss of cell surface E-cadherin is not associated with a concomitant increase in α-SMA expression and fibronectin production in PQ+SB431542-treated cells, providing evidence against EMT-like response in these cells. Essentially the same observations were obtained for cells treated with PQ for 12 days (Fig. 7A and 7B). Collectively, these results indicate the essential role of EMT-like cellular transformation in the production of fibronectin in PQ-treated cells, and that TGF-β1 signaling is required for these processes.

#### TGF-β signaling is required for the survival of A549 cells during low-dose long-term PQ exposure

**Fig 8:**
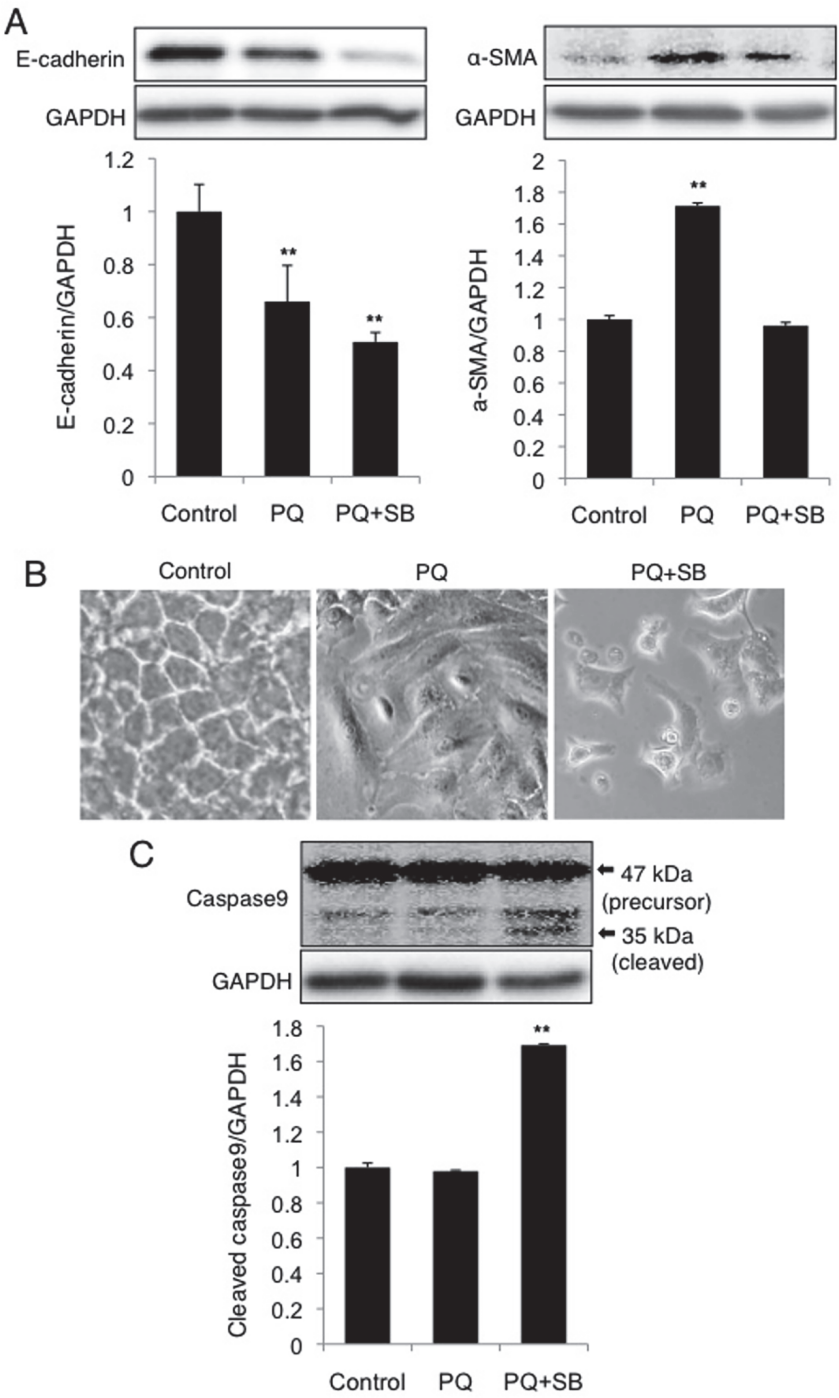
EMT-like response to low-dose PQ exposure is dependent on TGF-β1 signaling and is involved in the suppression of apoptosis. (A) SB431542 suppresses the increase in α-SMA but not the decrease in E-cadherin in PQ-treated cells. Following 12 days of exposure to 30 μM PQ with or without 10 μM SB431542, the cells were subjected to Western blot analysis. The levels of α-SMA and E-cadherin relative to GAPDH were determined by densitometric analysis (mean and SD, n = 3–4). The value of the control was set to 1. **p < 0.01 versus control. (B) Cytomorphology of A549 cells exposed to PQ with or without SB431542. (C) Increased activation of caspase9 in PQ+SB431542-treated cells. The cleaved form (35 kDa, indicated by the arrow) of caspase9 was detected by Western blot analysis. The levels of cleaved caspase9 relative to GAPDH are shown (mean and SD, n = 4). The value of the control was set to 1. **p < 0.01 versus control.

We evaluate the role of EMT-like response in the survival of A549 cells during low-dose long-term PQ exposure. In cells treated with PQ+SB431542 (12 days), α-SMA levels did not increase although the E-cadherin level decreased (Fig. 8A). These results are in agreement with the results of immunofluorescence analysis (Figs. 6 and 7), providing further evidence against EMT-like response in PQ+SB431542-treated cells. Light microscopic analysis showed the occurrence of substantial cell death in these cells (Fig. 8B). Indeed, the active form of caspase9 was significantly increased in PQ+SB431542-treated cells as compared to PQ-treated cells (Fig. 8C). Taken together, the inhibition of TGF-β1 signaling results in the suppression of EMT-like response, which makes it impossible for the cells to avoid apoptotic death.

#### Low-dose long-term PQ exposure induces EMT-like response, collagen secretion, and cell death avoidance via TGF-β1 signaling in NHBE cells

**Fig 9:**
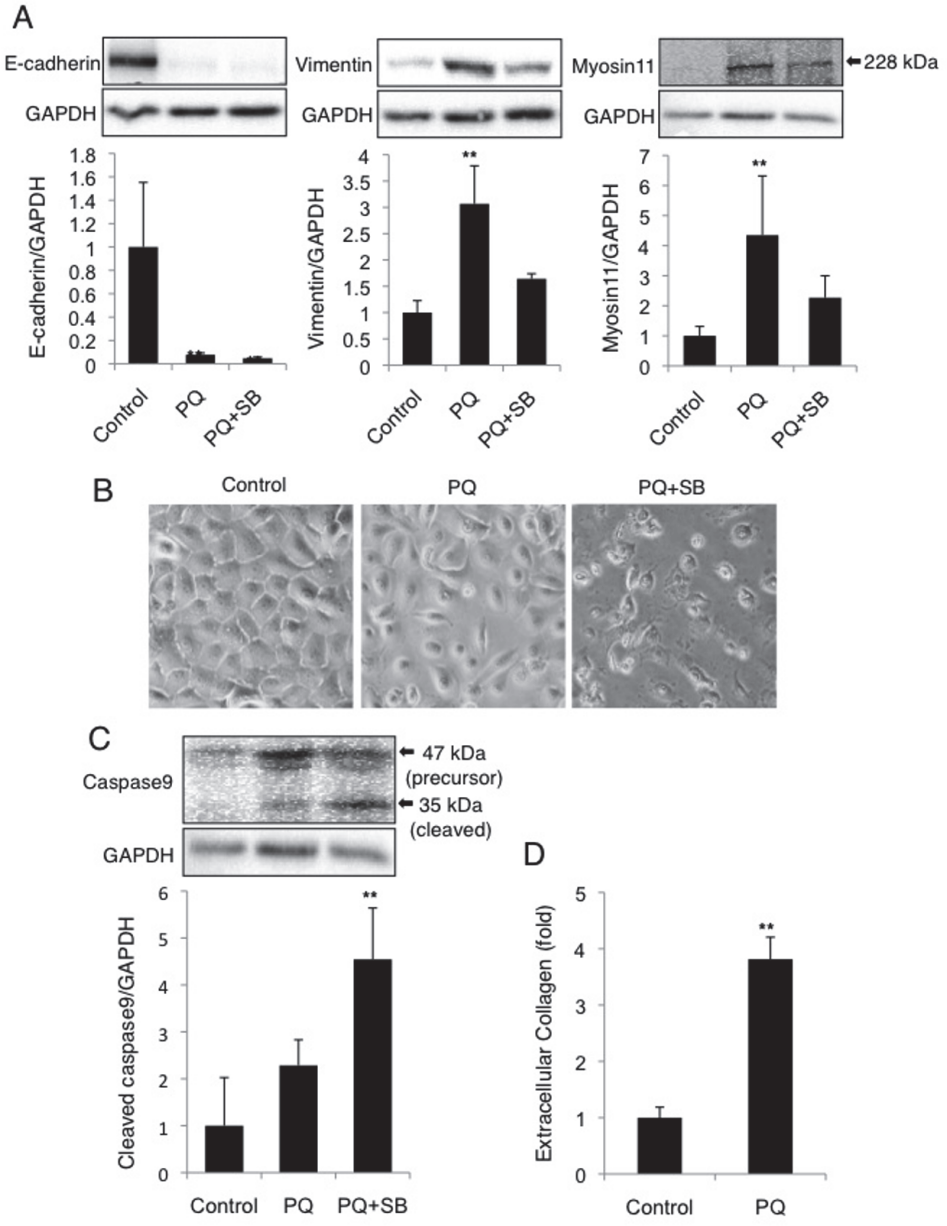
EMT-like response, increased secretion of collagen, and cell death avoidance in normal human bronchial epithelial cells subjected to low-dose long-term PQ exposure. Normal human bronchial epithelial (NHBE) cells were exposed to 30 μM PQ with or without 10 μM SB431542 for 12 days. (A) The decrease of E-cadherin and increase of vimentin and myosin11 in PQ-treated cells. Levels of these proteins were determined by Western blot analysis and normalized to GAPDH (mean and SD, n = 3–4). (B) Cytomorphology of NHBE cells exposed to PQ (30 μM for 12 days) with or without SB431542. (C) Increased activation of caspase9 in PQ+SB431542-treated cells. The cleaved form (35 kDa, indicated by the arrow) of caspase9 was detected by Western blot analysis. The levels of cleaved caspase9 relative to GAPDH are shown (mean and SD, n = 4). (D) Secretion of collagen from PQ-exposed NHBE cells. Levels of soluble collagen in the conditioned culture medium (10–12 days with or without 30 μM PQ) were examined by Sircol collagen assay. The value of the control was set to 1. **p < 0.01 versus control.

Finally we evaluated whether EMT-like response, fibrogenesis, and cell death avoidance are also occurred in normal pulmonary epithelial cells. Normal human bronchial epithelial (NHBE) cells were used for this purpose. Low-dose long-term PQ exposure (30 μM, 12 days) to NHBE cells resulted in the decrease of E-cadherin, increase of vimentin and myosin11 (smooth muscle myosin heavy chain, a mesenchymal marker), and morphological change into spindle-like shape (Fig. 9A and 9B), indicating EMT-like response in PQ-exposed NHBE cells. Co-administration of SB431542 not only attenuated the increase of mesenchymal marker protein (Fig. 9A) but also resulted in apoptotic cell death (Fig. 9B and 9C), confirming EMT-like response and cell death avoidance via TGF-β1 signaling in NHBE cells. Extracellular levels of collagen were also increased after PQ exposure, indicating that fibrogenesis was also occurred in PQ-exposed NHBE cells (Fig. 9D).

## Discussion

Regardless of treatment, most patients who ingest PQ die within a few days from multi-organ failure that includes acute renal failure, hepatic failure, myocardial damage and pulmonary edema. These consequences should be due, at least in part, to tissue destruction via cell death. In various experimental models, cell death caused by high-dose PQ exposure has been proposed to be apoptosis [21, 29, 30]. Our current results showing that high-dose (300 and 500 μM) short-term (2 days) exposure to PQ results in the apoptotic cell death of A549 cells (Fig. 1) is in agreement with previous reports [20, 21], further confirming the involvement of apoptosis in acute PQ injury. Clinical management of the acute phase of PQ intoxication is expected to improve, and several therapeutic trials to enhance PQ elimination have been reported to result in improved outcomes [31, 32]. However, patients who survive the acute phase of PQ intoxication often suffer delayed progressive pulmonary fibrosis, raising another problem concerning medications against PQ intoxication.

To the best of our knowledge, the present report is the first to show that PQ induces the secretion of an ECM protein through EMT-like cellular response from pulmonary cells. It has been reported in a rat model that PQ induces EMT during pulmonary fibrosis [17]. However, the relationship between EMT and pulmonary fibrosis during PQ poisoning has not been established to date due to the difficulty of identifying cells that secrete ECM proteins in response to PQ intoxication *in vivo*. Taking advantage of an *in vitro* system, we provide clear evidence that pulmonary epithelial cells secrete fibronectin or collagen via EMT-like cellular response: we observed EMT-like response in A549 and NHBE cells during low-dose (30 μM) long-term (6–12 days) PQ exposure (Figs. 3 and 9), and increased ECM proteins were detected in the culture medium after 12 days of exposure (Figs. 5A and 9D). Immunofluorescence analysis also revealed that the gain of a fibroblast phenotype, the production of fibronectin, is associated with the loss of cell surface E-cadherin as well as increased α-SMA expression in PQ-treated A549 cells (Figs. 6 and 7).

Significant increase of extracellular TGF-β1 levels was detected after 12 days exposure to 30 μM PQ (Fig. 5B), suggesting that PQ induces TGF-β1 secretion in A549 cells. SB431542 attenuates the expression of the precursor form of the TGF-β1 protein (Fig. 5C). This observation might suggest auto-induction of TGF-β1, which is observed in malignant cells and is mediated via elevated activation of Erk in these cells [33]. Taken together, we confirm the necessity of TGF-β1 signaling in EMT-like cellular response and subsequent fibrogenesis in PQ-treated pulmonary cells.

It is noteworthy that the treatment of A549 and NHBE cells to PQ+SB431542 resulted not only in the suppression of EMT-fibrogenesis, but also in apoptotic cell death (Figs. 8 and 9). It has been reported that EMT is required for epithelial cells to survive stressful situations that ordinarily lead to epithelial cell death. Anoikis is a specific type of apoptotic cell death observed especially in epithelial cells during the loss of cell attachment to ECM, and/or the loss of cell-to-cell adherence [10]. Anoikis contributes to proper epithelial cell turnover and the prevention of dysplastic tissue growth *in vivo* [10]. The oncogenic transformation of epithelial cells entails resistance to anoikis, which is achieved via EMT [22]. Snail, Twist and ZEB1 are proposed to be the transcriptional drivers of TGF-β-induced EMT [9] that bestow resistance to anticancer agents on epithelial cells. The suppression of such transcriptional factors render cells sensitive to anoikis [34, 35]. Our results indicating nuclear translocation of ZEB1 and Twist in response to PQ exposure (Fig. 4) suggest that the activation of these transcription factors should be involved in EMT-like response. Moreover, our results that the suppression of the TGF-β1-EMT axis of cellular transformation results in enhanced susceptibility to apoptotic cell death (Figs. 8 and 9) indicate the essential role of EMT-like response for maintaining cellular homeostasis against PQ toxicity.

The control of TGF-β signaling is a promising therapy against tissue fibrosis. Idiopathic pulmonary fibrosis (IPF) has recently been suggested to include EMT via TGF-β1 signaling. Anti-fibrotic agents such as pirfenidone, which have been shown to have considerable efficacy in the treatment of IPF in an experimental animal model [36], have been suggested to inhibit TGF-β1 signaling. However, our data showing that the inhibition of TGF-β1 signaling might accompany there-emergence of PQ toxicity, probably due to the loss of resistance to anoikis, indicates that caution should be exercised when attempting IPF treatments that involve the modulation of TGF-β1 signaling.

In conclusion, we present evidence that short-term high-dose PQ exposure leads to pulmonary cell death while long-term low-dose PQ exposure induces EMT-like cellular transformation and subsequent fibrogenesis in A549 and NHBE pulmonary epithelial cells. These results should be important to understand the mechanism of the delayed progressive pulmonary fibrosis observed during PQ intoxication in humans. EMT may be a process involved in protecting pulmonary cells against PQ-toxicity.
